# Endophytic Bacterial Communities Associated with Roots and Leaves of Plants Growing in Chilean Extreme Environments

**DOI:** 10.1038/s41598-019-41160-x

**Published:** 2019-03-20

**Authors:** Qian Zhang, Jacquelinne J. Acuña, Nitza G. Inostroza, María Luz Mora, Sergio Radic, Michael J. Sadowsky, Milko A. Jorquera

**Affiliations:** 1 The BioTechnology Institute, University of Minnesota, 140 Gortner Lab, 1479 Gortner Ave., St Paul, MN 55108-6106 USA; 2 Laboratorio de Ecología Microbiana Aplicada (EMAlab), Departamento de Ciencias Químicas y Recursos Naturales, Universidad de La Frontera, Ave. Francisco Salazar 01145, Temuco, Chile; 3 Network for Extreme Environment Research (NEXER), Scientific and Technological Bioresource Nucleus (BIOREN), Universidad de La Frontera, Ave. Francisco Salazar 01145, Temuco, Chile; 4 Departamento de Ciencias Agropecuarias y Acuícolas, Universidad de Magallanes, Ave. Bulnes 01855, Punta Arenas, Chile; 5 Department of Soil, Water, and Climate, and Department of Plant and Microbial Biology, University of Minnesota, 439 Borlaug Hall, 1991 Upper Buford Circle, St. Paul, MN 55108 USA

## Abstract

Several studies have demonstrated the relevance of endophytic bacteria on the growth and fitness of agriculturally-relevant plants. To our knowledge, however, little information is available on the composition, diversity, and interaction of endophytic bacterial communities in plants struggling for existence in the extreme environments of Chile, such as the Atacama Desert (AD) and Patagonia (PAT). The main objective of the present study was to analyze and compare the composition of endophytic bacterial communities associated with roots and leaves of representative plants growing in Chilean extreme environments. The plants sampled were: *Distichlis spicate* and *Pluchea absinthioides* from the AD, and *Gaultheria mucronata* and *Hieracium pilosella* from PAT. The abundance and composition of their endophytic bacterial communities was determined by quantitative PCR and high–throughput sequencing of 16S rRNA, respectively. Results indicated that there was a greater abundance of 16S rRNA genes in plants from PAT (10^13^ to 10^14^ copies g^−1^ DNA), compared with those from AD (10^10^ to 10^12^ copies g^−1^ DNA). In the AD, a greater bacterial diversity, as estimated by Shannon index, was found in *P*. *absinthioides*, compared with *D*. *spicata*. In both ecosystems, the greater relative abundances of endophytes were mainly attributed to members of the phyla *Proteobacteria* (14% to 68%), *Firmicutes* (26% to 41%), *Actinobacteria* (6 to 23%) and *Bacteroidetes* (1% to 21%). Our observations revealed that most of operational taxonomic units (OTUs) were not shared between tissue samples of different plant species in both locations, suggesting the effect of the plant genotype (species) on the bacterial endophyte communities in Chilean extreme environments, where *Bacillaceae* and *Enterobacteriacea* could serve as keystone taxa as revealed our linear discriminant analysis.

## Introduction

Numerous studies have revealed that bacteria living within plant tissues, collectively called endophytic bacteria, play a crucial role in the growth and fitness of a wide variety of monocot and dicot plant species, among others^1,2^. Beneficial functions attributed to endophytic bacteria include plant growth promotion by supplying nutrients (*e*.*g*., nitrogen fixation), protection against biotic- (*e*.*g*., pathogens) and abiotic-stresses (*e*.*g*., salinity and drought), detoxification of harmful compounds (*e*.*g*., NH_3_ or CN), and the production of bioactive compounds (*e*.*g*., secondary metabolites and hormones)^3,4^. Various endophytic microorganisms have been categorized as plant growth–promoting bacteria (PGPB) and they are currently used in the formulation of diverse bioproducts (*e*.*g*., biofertilizers and biofungicides) or to modify and/or introduce beneficial bacteria into the plant phytomicrobiome for agricultural purposes^5,6^. To date, however, many microbiome studies have been done using model plant (*e*.*g*., *Arabidopsis thaliana*), commercially relevant plants for agriculture (*e*.*g*., wheat, soybean, rice, maize, etc.) and wild plant species (*e*.*g*., weeds and trees) grown under laboratory, greenhouse and fields conditions^1,2,7,8^. Consequently, we only have limited knowledge on the composition and interactions of microbiota and plants, especially endophytic bacterial communities, on native plant vegetation growing in extreme environments, such as hot and/or cold deserts. Thus, our understanding on microbial interactions in plant holobiont will be key in the develop of efficient strategies for native plant conservation and/or exploit the full yield potential of crop plants under climate change scenario^9^.

The country of Chile is long (4,270 km) and narrow (mean width 177 km) and harbors a great variety of pristine ecosystems. The Atacama Desert (AD) is located in the northern region of Chile (from 18°24′S to 29°55′S) and is considered among the driest places on earth. In contrast, the Chilean Patagonia (from 41°08S to 56°30′S) is located in the far south of the country and is a sub Antarctic region. Both regions have extreme environments and their plant-associated bacterial communities have been barely studied thus far. In this context, we have reported that members of the orders *Enterobacteriales*, *Actinomycetales*, and *Rhizobiales* comprise dominant groups of the bacterial communities in the rhizosphere (the soil influenced by plant roots) of shrubs grown in AD and Patagonia (PAT), namely *Atriplex* sp. and *Chuquiraga* sp., respectively^10^. Results of this study also suggested that some isolates, belonging to the genera *Enterobacteria*, *Pseudomonas*, and *Bacillus*, were putative PGPB. The ability of the native isolates from AD to act as PGPB was confirmed by formulation and inoculation of a bacterial consortium onto plants. These studies revealed that wheat plants inoculated with the consortium produced greater biomass under water shortage and field conditions, compared with uninoculated seedlings^11^. A recent study also showed a greater protection against salt stress in wheat plants inoculated with rhizosphere bacteria isolated from Andean Altiplano native plant (*Parastrephia quadrangularis*) in AD^12^. However, these studies did not take into account the composition and interaction of native endophytic bacteria in Chilean extreme environments, as well as their potential use as PGPB.

During the last several years, advances in high–throughput DNA sequencing (HTS) technologies (*e*.*g*., Illumina®, PacBio® and Oxford Nanopore®) have opened new windows into the microbial ecology of a variety of environments, allowing the detailed study of complex bacterial communities in nature as never seen before. Thus, HTS platforms have widely been used to decipher the structure and function of microbiota in different compartments of plants, including as the rhizosphere, endosphere (inner tissues of plants), and phyllosphere (the aerial part of plant leaves)^1,13^. Results of 454‒pyrosequencing studies showed that the *Proteobacteria* (mainly *Gammaproteobacteria*) were the dominant taxa in the rhizospheres of *Atriplex* sp. and *Stipa* sp. (shrubs) grown in the AD^14^. These authors also postulated that native plants from Chilean extreme environments may attract, select, and conserve specific bacterial groups in order to sustain plant growth and tolerance to local harsh conditions. Based on this supposition, the main goal of the present study was to describe and compare the relative abundances and composition of bacterial communities associated with roots and leaves of plants grown in the AD and PAT regions of Chile by using HTS of 16S rRNA genes.

## Material and Methods

### Sampling

**Figure 1:**
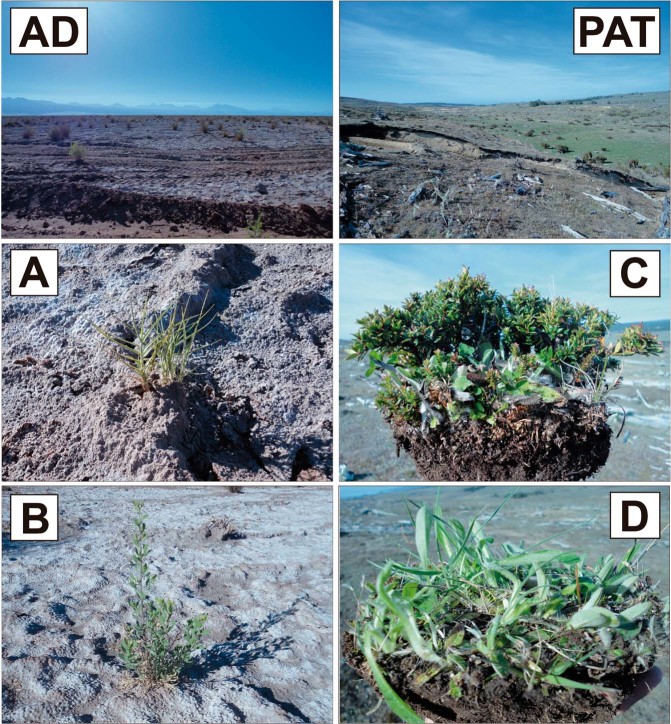
Sampling of plants from (AD) Atacama Desert (*Distichlis spicata* (**A**) and *Pluchea absinthioides* (**B**)), and (PAT) Patagonia (*Gaultheria mucronata* (**C**) and *Hieracium pilosella* (**D**)). Three specimens per plant species were taken in each location from a 10 m transect.

Plant specimens were collected in the AD (23°1′59″S, 68°11′59″W; and PAT (53°28′0″S, 71°0′59″W) regions of Chile (Fig. 1). The plants sampled were: *Distichlis spicate* (Poacea; Fig. 1A) and *Pluchea absinthioides* (Asteraceae; Fig. 1B) from AD, and *Gaultheria mucronata* (Ericaceae; Fig. 1C), and *Hieracium pilosella* (Asteraceae; Fig. 1D) from PAT. Three specimens of each plant species were randomly taken in a 10 m transect by using a clean spade to remove intact roots from soil. Specimens were placed in plastic bags and immediately transported, on ice, to the Applied Microbial Ecology Laboratory at La Frontera University for microbiological analyses.

### DNA extraction

Roots and leaves samples were separated and surface sterilized by repeated immersion in 70% (v/v) ethanol for 3 min, followed by 2.5% (v/v) sodium hypochlorite (NaOCl) for 5 min as described by Barra *et al*.^15^. Roots were exhaustively rinsed with sterile distilled water. Triplicate portions of roots and leaves were aseptically cut, frozen in liquid nitrogen, macerated and homogenized with a mortar and pestle, and stored at −80 °C until DNA extraction. Samples of the homogenized tissues (0.25 g) were used for DNA extraction with Quick‒DNA^TM^ Plant/seed Miniprep kits according to manufacturer instructions (Zymo Research, CA, USA). The quantity and purity of DNA extracts were determined by measuring absorbance at 260 nm and 280 nm by using a microplate spectrophotometer (Multiskan GO, Thermo Fisher Scientific, Inc., MA, USA).

### Quantitative PCR

The abundance of endophytic bacteria in each tissue sample was determined by quantitative PCR (qPCR) by using a universal primer set for the bacterial 16S rRNA gene (Bac1369F 5′-CGG TGA ATA CGT TCY CGG-3′) and Prok1492R (5′-GGW TAC CTT GTT ACG ACT-3′) as previously described^16,17^. Briefly, PCR conditions were run with an enzyme activation step at 95 °C for 10 min, followed by 40 cycles of 15 s at 95 °C, and 1 min of annealing plus extension at 60 °C. PCR reactions were performed in triplicate per plant species (including technical triplicates) with 20 µg L^−1^ of total DNA in a StepOnePlus^TM^ Real–Time PCR System (Applied Biosystems, Inc., CA, USA) using PowerUp^TM^ SYBR^TM^ Green Master Mix (Applied Biosystems, Inc.), by following the manufacturer instructions. The numbers obtained were normalized and analyzed by using one‒way ANOVA, and comparisons were done by using Tukey’s post‒hoc test. Differences were considered to be significant when the *P* value was ≤0.05.

### High‒Throughput DNA Sequencing

The distribution and relative abundances of endophytic bacteria in root and leaf tissues was assessed by HTS using triplicate samples of each plant species as follow. The V4 hypervariable region of the 16S rRNA genes were amplified, for bacteria and archaea, by using primer set 515F (5′-GTG CCA GCM GCC GCG GTA A-3′) and 806R (5′-GGA CTA CHV GGG TWT CTA AT-3′). Sequencing was done by the University of Minnesota Genomics Center (UMGC, Minneapolis, MN, USA)^18^ using barcode primers and the dual indexing method. Amplicons were gel purified, pooled, and paired‒end sequenced at a read length of 300 nt on the Illumina MiSeq platform (Illumina, Inc., San Diego, CA, USA) from UMGC.

### Bioinformatics and Statistical Analysis

Sequences were analyzed by using mothur program ver. 1.34.0 (https://www.mothur.org)^19^. The first 150 nt were trimmed from sequences to remove low–quality regions at the ends of reads. Fastq–join software was used to join paried–end sequencing reads^20^, the joined sequencing reads were trimmed to maintain an average quality score >35, a homopolyer length >8 nt. Sequences with >2 mismatches in primer sequences and ambiguous bases were removed. High quality sequencing reads were aligned on the basis of the SILVA database ver. 123^21^, and subjected to a 2% pre–clustering step to remove possible sequence errors^22^. The UCHIME software was used to identify and remove probable chimeric sequences^23^. To avoid the influence of non-microbiota (e.g., Chloroplast and mitochondria), the sequences were futher filtered by Qiime to remove non-microbiota taxa before a subsequent analysis. Sequence data was rarefied to 700 and 4,500 sequence reads per data set prior to statistical analysis for AD and PAT, respectively. Raw sequencing data were deposited in the Sequence Read Archive (SRA) of NCBI under Accession Number SRP156290.

For statistical analysis, Alpha diversity indices, as well as Good’s coverage, were calculated using the Shannon index and the abundance–based coverage estimate (ACE) through the mothur program. Visualization of the taxonomic distribution of microbial communities was performed using the “ggplot2” package in R^24^. Differences in beta diversity was evaluated by using analysis of similarity (ANOSIM) and permutational multivariate analysis of variance (PERMANOVA). Principal coordinate analysis (PCoA) was performed based on unweighted unifrac distance for the ordination^25^. The VennDiagram package in R was used to identify shared OTUs of endophytic bacterial communities between root and leave tissues^26^. Variations in taxa associating with root and leaf tissues were evaluated using linear discriminant analysis (LDA) of effect sizes^27^, which employs Kruskal–Wallis and Wilconxon rank–abundance tests and then utilizes linear discriminant analysis (LDA) to estimate effect sizes of the features.

## Results

### Abundances of Bacteria

In general terms, plant tissues from Patagonia (*G*. *mucronata* and *H*. *pilosella*) had greater abundances of bacteria (from 10^13^ to 10^14^ 16S rRNA gene copies g^−1^ template DNA), compared with those from AD (*D*. *spicata* and *P*. *absinthioides*) (from 10^10^ to 10^12^ 16S rRNA genes copies g^−1^ template DNA), except roots from *G*. *mucronata* (Table 1). In AD plants, a significantly (*P* ≤ 0.05) greater abundance of bacteria in both tissues was found in *P*. *absinthioides* (2.6 × 10^11^ and 2.7 × 10^12^ 16S rRNA gene copies g^−1^ template DNA in roots and leaves, respectively) compared with those from *D*. *spicata* (4.4 × 10^10^ and 5.4 × 10^11^ 16S rRNA gene copies g^−1^ template DNA in roots and leaves, respectively). In PAT plants, a significantly (*P* ≤ 0.05) greater abundance of bacteria in both tissues was found in *H*. *pilosella* (8.1 × 10^13^ and 5.4 × 10^14^ 16S rRNA gene copies g^−1^ template DNA in roots and leaves, respectively) compared with those from *G*. *mucronata* (1.3 × 10^13^ and 3.0 × 10^10^ 16S rRNA gene copies g^−1^ template DNA in roots and leaves, respectively).

**Table 1:** Counts of total bacteria in root and leaf tissues among plants from Atacama Desert (*Distichlis spicata* and *Pluchea absinthioides*) and Patagonia (*Gaultheria mucronata* and *Hieracium pilosella*) determined by quantitative PCR (qPCR).

Location	Plant tissues	Plant specie	Counts^†^ (16S rRNA genes copies g^−1^ DNA)
Atacama Desert	Roots	*D*. *spicata*	4.4 ± 0.4 × 10^10 C*^
		*P*. *absinthioides*	2.6 ± 0.6 × 10^11 B^
	Leaves	*D*. *spicata*	5.4 ± 1.2 × 10^11 B^
		*P*. *absinthioides*	2.7 ± 0.8 × 10^12 A^
Patagonia	Roots	*G*. *mucronata*	1.3 ± 0.2 × 10^13 C^
		*H*. *pilosella*	8.1 ± 0.4 × 10^13 B^
	Leaves	*G*. *mucronata*	3.0 ± 1.4 × 10^10 D^
		*H*. *pilosella*	5.4 ± 1.1 × 10^14 A^

^†^The values represent mean ± standard error from *n* = 3.

^*^Sample groups sharing the same letter did not vary significantly (*P* ≤ 0.05) by ANOVA followed by Tukey’s post-hoc test.

### Composition of Endophytic Bacterial Community in Extreme Environments

Sequence analyses showed a lower estimated coverage in AD (from 91 to 95%) compared with PAT (from 98 to 99%) (Table 2). The values of observed OTUs (S_obs_) were lower in plant tissues from AD compared with those from PAT, ranging in values from 68 to 113 and 208 to 220 in roots, and 103 to 152 and 151 to 160 in leaves, respectively. Similarly, lower ACE values were also observed in AD compared to PAT plants, ranging in values of 148 to 188 and 263 to 314 in roots, and 139 to 211 and 190 to 245 in leaves, respectively. However, and as revealed by the Shannon index, there was significant differences (Tukey’s post‒hoc test, *P* < 0.05) in bacterial diversity in both tissues of *P*. *absinthioides* compared with tissues from *D*. *spicata* in AD (Table 2). In contrast, no significant differences in bacterial diversity were found in plant tissues from PAT plants.

**Table 2:** Coverage and alpha diversity among endophytic bacterial communities in root and leaf tissues of plants from Atacama Desert (*Distichlis spicata* and *Pluchea absinthioides*) and Patagonia (*Gaultheria mucronata* and *Hieracium pilosella*), based on high‒throughput DNA sequencing data and analyzed by mothur program.

Location	Plant tissues	Plant specie	Coverage (%)	S_obs_^†^	Shannon index	ACE^‡^
Atacama Desert	Roots	*D*. *spicata*	95.429 ± 1.51^A^	68 ± 13^A^	2.28 ± 0.21^A^	148 ± 73^A^
		*P*. *absinthioides*	93.952 ± 3.48^A^	113 ± 39^A^	3.78 ± 0.71^B^	188 ± 122^A^
	Leaves	*D*. *spicata*	91.905 ± 1.51^A^	152 ± 24^A^	4.41 ± 0.26^A^	211 ± 36^A^
		*P*. *absinthioides*	95.143 ± 0.49^B^	103 ± 8^B^	3.92 ± 0.1^B^	139 ± 11^B^
Patagonia	Roots	*G*. *mucronata*	98.61 ± 0.26^A^	208 ± 41^A^	3.93 ± 0.88^A^	314 ± 49^A^
		*H*. *pilosella*	98.94 ± 0.21^A^	220 ± 14^A^	4.41 ± 0.13^A^	263 ± 28^A^
	Leaves	*G*. *mucronata*	98.83 ± 0.45^A^	151 ± 94^A^	2.76 ± 1.61^A^	245 ± 120^A^
		*H*. *pilosella*	99.24 ± 0.26^A^	160 ± 42^A^	4.02 ± 0.27^A^	190 ± 52^A^

^†^*S*_obs_: number of OTUs observed at 97% similarity.

^‡^ACE: abundance-based coverage estimate.

^*^The values represent mean ± standard deviation from *n* = 3.

^**^Sample groups sharing the same letter in each tissue did not vary significantly (*P* ≤ 0.05) by ANOVA followed by Tukey’s post-hoc test.

**Figure 2:**
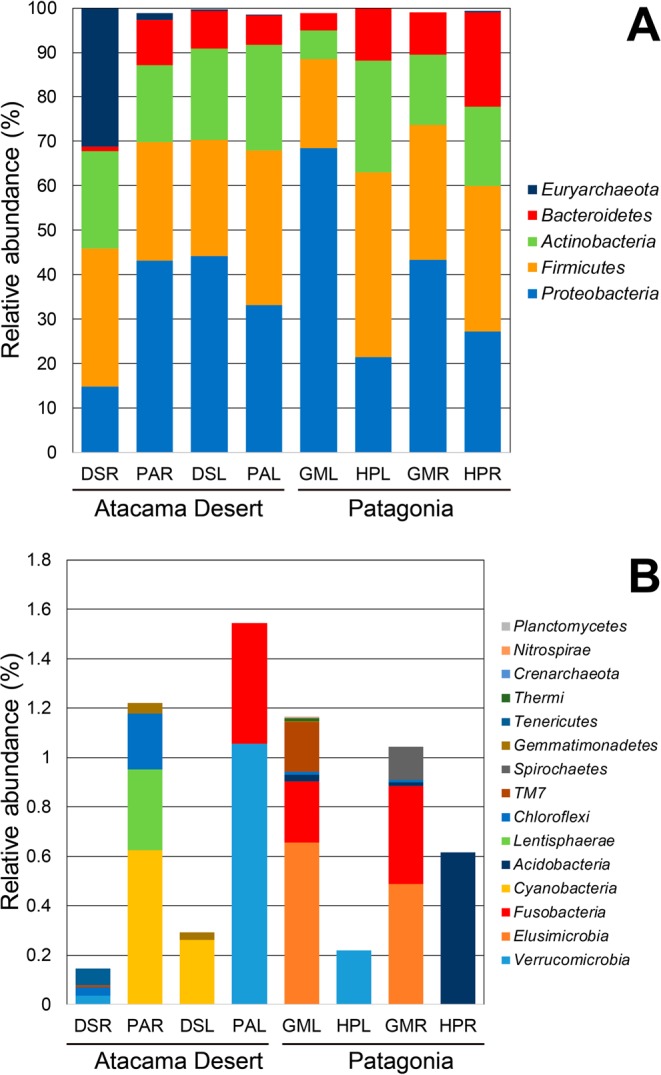
Mean relative abundances of major (**A**) and minor (**B**) taxa (at the phylum level) among endophytic bacterial communities from plant root and leaf tissues obtained from the Atacama Desert (*Distichlis spicata* and *Pluchea absinthioides*) and Patagonia (*Gaultheria mucronata* and *Hieracium pilosella*). Legend: DSR: root tissue from *D*. *spicata*, PAR: root tissue of *P*. *absinthioides*, DSL: leaf tissues from *D*. *spicata*, PAL: leaf tissues from *P*. *absinthioides*, GMR: root tissue from *G*. *mucronata*, HPR: root tissue from *H*. *pilosella*, GML: leaf tissues from *G*. *mucronate*, and HPL: leaf tissues from *H*. *pilosella*. Values represent means of 3 replicates.

In both ecosystems, the assignment of taxonomic affiliation to endophytic bacterial communities at the phylum level indicated that there were high relative abundances of *Proteobacteria* (14.88% to 68.53%), *Firmicutes* (26.03% to 41.59%), *Actinobacteria* (6.45% to 23.69%), and *Bacteroidetes* (1.09% to 21.21%) in both tissues (Fig. 2A). It is noteworthy that the lowest relative abundance of *Bacteroidetes* was found in roots from *D*. *spicata*. This tissue, however, presented a relative abundance of 31.01% of members belonging to *Euryarchaeota* phylum (Fig. 2A).

**Figure 3:**
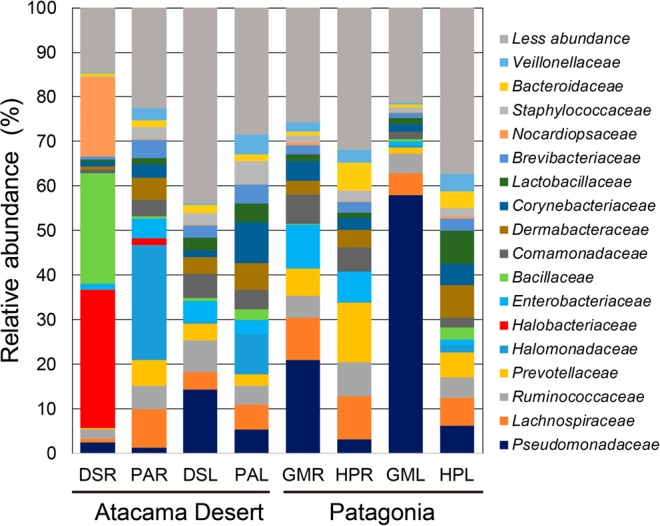
Mean relative abundances of taxa (at the family level) among endophytic bacterial communities in root and leaf tissues obtained from plants in the Atacama Desert (*Distichlis spicata* and *Pluchea absinthioides*) and Patagonia (*Gaultheria mucronata* and *Hieracium pilosella*). Legend: DSR: root tissue from *D*. *spicata*, PAR: root tissue of *P*. *absinthioides*, DSL: leaf tissues from *D*. *spicata*, PAL: leaf tissues from *P*. *absinthioides*, GMR: root tissue from *G*. *mucronata*, HPR: root tissue from *H*. *pilosella*, GML: leaf tissues from *G*. *mucronate*, and HPL: leaf tissues from *H*. *pilosella*. Values represent means of 3 replicates.

With respect to minor taxa, broad taxonomic diversity among samples was found. The tissues of AD plants (*D*. *spicata* and *P*. *absinthioides*) showed large relative abundances of *Cyanobacteria*, *Lentisphaerae* and *Chloroflexi* in roots, and *Verrucomicrobia* and *Fusobacteria* and *Cyanobacteria* in leaves (Fig. 2B). The tissues from PAT plants (*G*. *mucronata* and *H*. *pilosella*) showed a large relative abundance of *Elusimicrobia*, *Fusobacteria*, *Spirochaetes* and *Acidobacteria* in roots, and *Elusimicrobia*, *Fusobacteria*, TM7 and *Verrucomicrobia* in leaves (Fig. 2B). At family level, a wide taxonomic diversity among samples was also found. In AD plants, higher relative abundances of *Halobacteriaceae* (31.01%), *Bacillaceae* (24.67%) and *Nocardiopsaceae* (17.78%) are highlight in roots of *D*. *spicata* whereas a higher relative abundance of *Halomonadaceae* (25.81%) is highlight in leaves of *P*. *absinthioides* (Fig. 3). In PAT plants, a higher relative abundance of members belonging to *Pseudomonaceae* was found in roots (21%) and leaves (57.93%) from *G*. *mucronata*.

**Figure 4:**
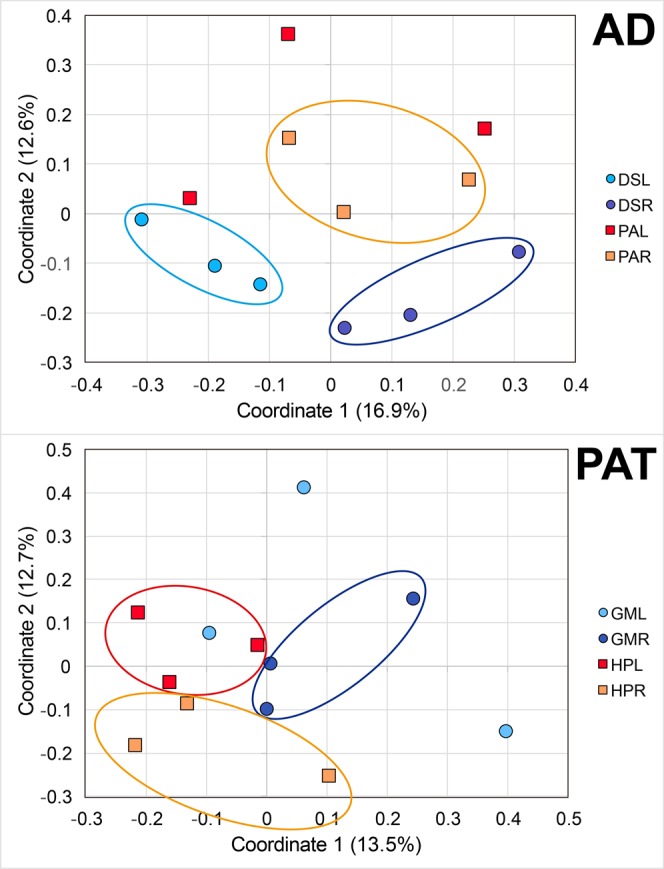
Principal coordinate analysis (PCoA) of endophytic bacterial communities from plant roots and leaf tissues obtained from the (AD) Atacama Desert (*Distichlis spicata* and *Pluchea absinthioides*), and (PAT) Patagonia (*Gaultheria mucronata* and *Hieracium pilosella*). Legend: DSR: root tissue from *D*. *spicata*, PAR: root tissue of *P*. *absinthioides*, DSL: leaf tissues from *D*. *spicata*, PAL: leaf tissues from *P*. *absinthioides*, GMR: root tissue from *G*. *mucronata*, HPR: root tissue from *H*. *pilosella*, GML: leaf tissues from *G*. *mucronate*, and HPL: leaf tissues from *H*. *pilosella*. Analyses were done by using unweighted UniFrac distances.

Differences between tissues and plant species were also confirmed by PCoA. In AD plants, a clear grouping between roots and leaves from *D*. *spicata*, and roots from *P*. *absinthioides* was observed (Fig. 4, AD). Similarly, a clear grouping between roots and leaves from *H*. *pilosella*, and roots from *G*. *mucronata* was also observed in PAT plants (Fig. 4, PAT).

### Shared and Unique Operational Taxonomic Units and Keytone Taxa in Extreme Ecosystems

**Figure 5:**
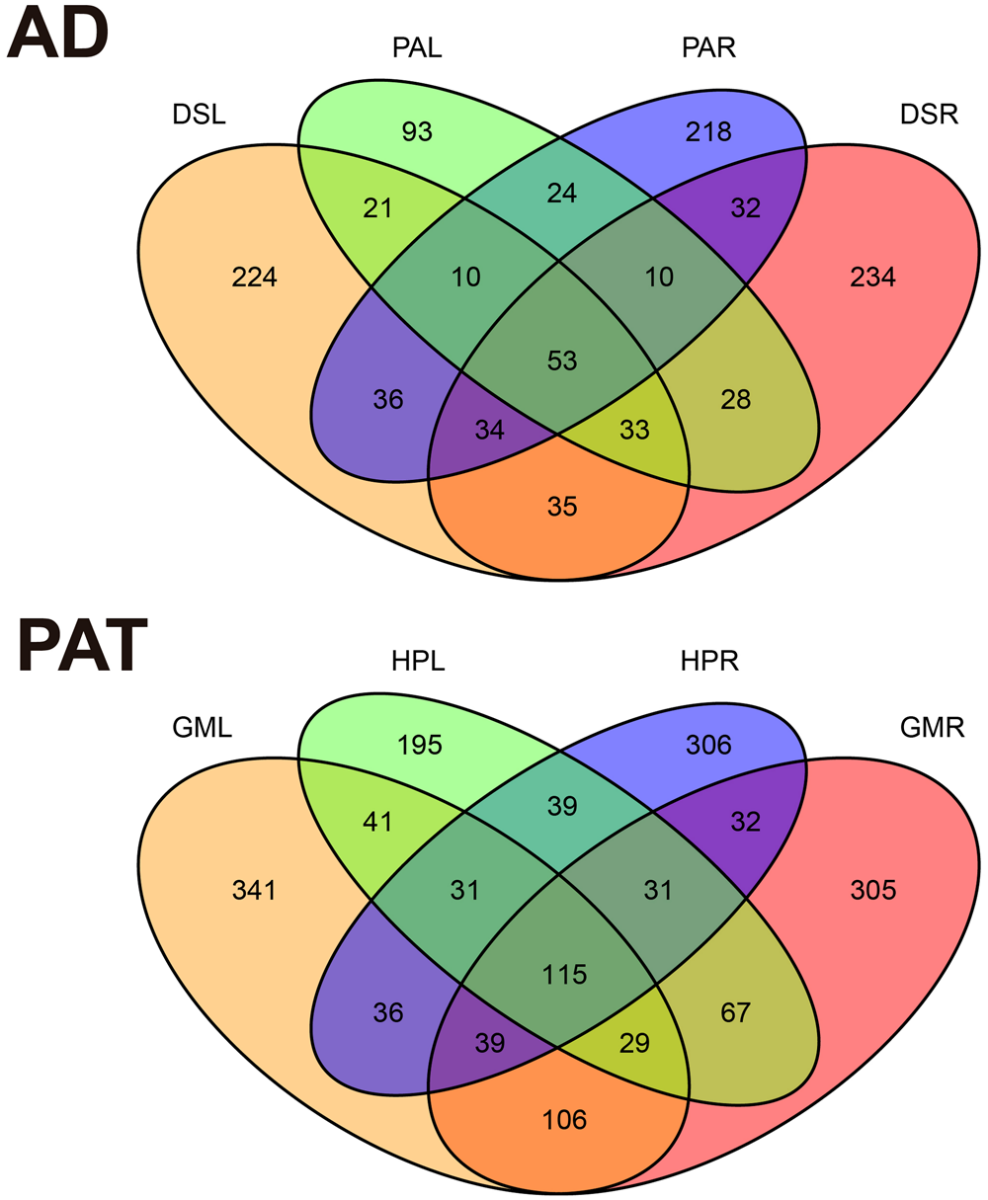
Shared OTUs among endophytic bacterial communities in roots and leaves of plants from (AD) Atacama Desert (*Distichlis spicata* and *Pluchea absinthioides*), and (PAT) Patagonia (*Gaultheria mucronata* and *Hieracium pilosella*) determined in each plant species (*n* = 3). Legend: DSR: root tissue from *D*. *spicata*, PAR: root tissue of *P*. *absinthioides*, DSL: leaf tissues from *D*. *spicata*, PAL: leaf tissues from *P*. *absinthioides*, GMR: root tissue from *G*. *mucronata*, HPR: root tissue from *H*. *pilosella*, GML: leaf tissues from *G*. *mucronate*, and HPL: leaf tissues from *H*. *pilosella*.

**Figure 6:**
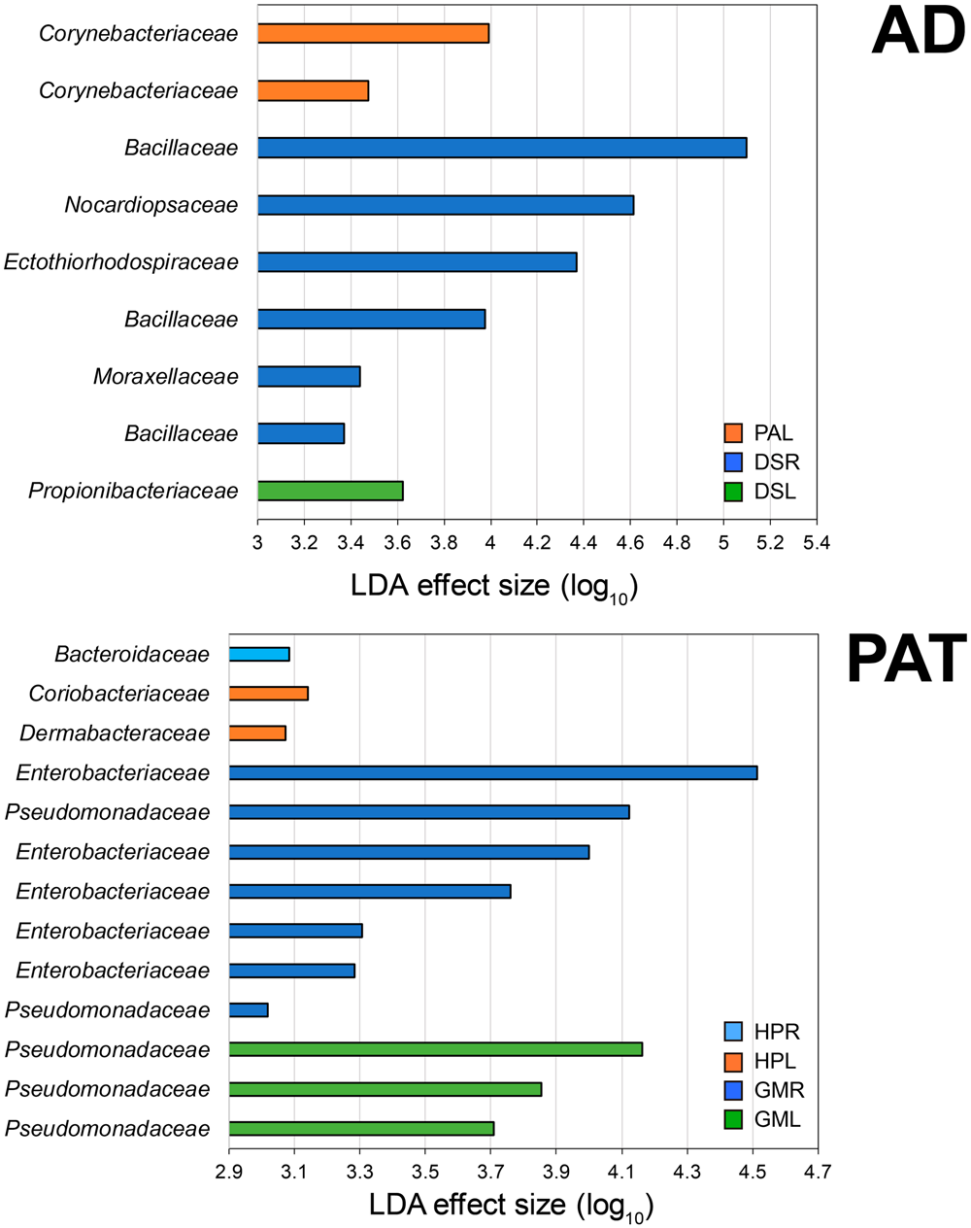
Key phylotypes of endophytic microbiota residing within roots and leaves of plants growing in the (AD) Atacama Desert and (PAT) Patagonia. Differences between plant compartments were assessed by using linear discriminant analysis (LDA) of effect size and Kruskal–Wallis and Wilconxon rank–abundance tests. The histogram shows the linear discriminant analysis scores, computed for features (at the operational taxonomic unit level), of differentially abundant taxa (at the family level) on root and leaf tissues of plants. Histogram bars representing the same bacterial families were deduced from different taxa. Legend: DSR: root tissue from *D*. *spicata*, PAR: root tissue of *P*. *absinthioides*, DSL: leaf tissues from *D*. *spicata*, PAL: leaf tissues from *P*. *absinthioides*, GMR: root tissue from *G*. *mucronata*, HPR: root tissue from *H*. *pilosella*, GML: leaf tissues from *G*. *mucronate*, and HPL: leaf tissues from *H*. *pilosella*.

In relation to the distribution of shared and unique OTUs among endophytic bacterial communities in ecosystems, 53 out of 1075 OTUs were shared in AD, while 115 out of 1713 OTUs shared in PAT ecosystem (Fig. 6 and Table 3). In AD plants, among these 53 shared OTUs, most belonged to the *Firmicutes* (20), followed by *Proteobacteria* (15) and *Actinobacteria* (13). In PAT plants, among these 115 shared OTUs, most belonged to the *Firmicutes* (37), followed by the *Actinobacteria* (29), *Bacteroidetes* (25) and *Proteobacteria* (24). In the PAT, there were a greater number of unique OTUs, relative to those seen at AD, where 305 and 341 and 306 and 195 plant specific OTUs were found in root and leaves from *G*. *mucronata* and *H*. *pilosella*, respectively (Table 3 and Fig. 5). In the AD, 234 and 224, and 218 and 93 plant specific OTUs were found in root and leaves from *D*. *spicata* and *P*. *absinthioides*, respectively (Table 3 and Fig. 5). In AD plants, most of unique OTUs belonged to the *Firmicutes* (353), followed by the *Proteobacteria* (213), *Actinobacteria* (90) and *Bacteroidetes* (87). Similarly, in PAT plants, most of unique OTUs belonged to the *Firmicutes* (512), followed by *Proteobacteria* (397), *Actinobacteria* (159) and *Bacteroidetes* (119) (Table 3).

**Table 3:** Distribution of shared and unique operational taxonomic units (OTUs) among endophytic bacterial communities in root and leaf tissues of plants from Atacama Desert (*Distichlis spicata* [DS] and *Pluchea absinthioides* [PA]) and Patagonia (*Gaultheria mucronata* [GM] and *Hieracium pilosella* [HP]), based on high‒throughput DNA sequencing data in each plant species (*n* = 3).

Location	Taxa (phylum)	Shared OTUs in ecosystems	Roots	Unique OTUs				Leaves	Unique OTUs			
			Shared OTUs					Shared OTUs				
				DS	PA	GM	HP		DS	PA	GM	HP
Atacama Desert	Actinobacteria	13	2	40	25			2	13	12		
	Bacteroidetes	4	2	25	23			2	30	9		
	Chloroflexi			1	1							
	Cyanobacteria				1				1			
	Euryarchaeota	1	1	8	1				1			
	Firmicutes	20	17	109	126			10	71	47		
	Fusobacteria									2		
	Gemmatimonadetes				1				1			
	Lentisphaerae				1							
	Proteobacteria	15	10	48	39			7	107	19		
	TM7			1								
	Tenericutes			1								
	Verrucomicrobia			1						4		
	Sub-total	53	32	234	218			21	224	93		
Patagonia	Acidobacteria					2	3				3	
	Actinobacteria	29	1			56	28	2			52	23
	Bacteroidetes	25	5			19	38	5			37	25
	Chloroflexi					1					5	
	Crenarchaeota										1	
	Euryarchaeota						2					
	Firmicutes	37	16			114	153	28			148	97
	Fusobacteria										5	
	Gemmatimonadetes										2	
	Nitrospirae										1	
	Planctomycetes										1	
	Proteobacteria	24	10			112	82	6			79	45
	Spirochaetes					1						
	Thermi										3	
	TM7										3	
	Verrucomicrobia										1	5
	Sub-total	115	32			305	306	41			341	195
Total		168	64	234	218	305	306	62	224	94	341	195

Linear discriminant analysis (LDA) of effect size was performed to determine which taxa varied among plant components at two ecosystems. Several bacterial taxa, which belong to the *Bacillacea*, *Nocardiopsaceae*, *Ectothiorhodospiraceae*, and *Moraxellaceae* (at family level), elucidative served as the keystone taxa in roots of *D*. *spicata*, while only the *Propionivacteriaceae* and *Corynebacteriaceae* could be used to indicate leaves of *D*. *spicata* and *P*. *absinthioides*, respectively (Fig. 6). Moreover, the *Bacillaceae* had the greatest effect size among the plant components in AD. Contrastingly, taxa belonging to the family level of the *Enterobacteriaceae*, *Pseudomonadaeceae*, *Dermabacteraceae*, *Coriobacteriaceae*, and *Bacteroidaceae* were the keystones presenting endophytic microbiota among plant compartments in PAT ecosystem (Fig. 6). Additionally, the *Enterobacteriaceae* had the highest effect size in PAT.

## Discussion

Microbial endophytes play a central role in the ecology, evolution, and growth promotion of plants^2,4,28^. However, despite their importance, there is scant knowledge concerning endophytic microbial populations in plants living in extreme environments, including Chile. Our study showed that the abundances of bacteria from the endosphere of root and leaf tissues ranged from 10^10^ to 10^12^ and from 10^10^ to 10^14^ 16S rRNA gene copies g^−1^ template DNA in AD and PAT, respectively; corresponding from 10^3^ to 10^7^ copies of 16S rRNA genes copies g^−1^ fresh tissue.

A wide range of endophytic prokaryote densities have been reported in tissues of different plants thus far. Similar to our finding, 10^14^ 16S rRNA gene copies g^−1^ template DNA of total endophytic prokaryotes were reported in endospheres of olive tree (*Olea europaea* L.) leaves collected from diverse Mediterranean ecosystems^29^. Higher abundances than those observed in this current study were reported to be present in the endosphere of rice (10^7^ to 10^8^ 16S rRNA gene copies g^−1^ root) and crops (10^10^ to 10^13^ copies of 16S rRNA genes g^−1^ root) by Ruppel *et al*.^30^ and Breidenbach *et al*.^31^, respectively. Under stress conditions, Blain *et al*.^32^ recently reported abundances of endophytic bacteria from 10^3^ to 10^5^ 16S rRNA gene copies g^−1^ from fresh roots of natural vegetation growing in a hydrocarbon‒contaminated site. In addition, and to our knowledge, there are no studies reporting the abundances of endophytic bacterial populations in plants from Chilean extreme environments. It should be noted, however that a previous study of the rhizosphere of plants from AD and PAT revealed values of 10^9^ and 10^11^ 16S rRNA genes copies g^−1^ of soil, respectively^33^.

With respect to analyses of alpha diversity of endophytic bacterial communities in root and leave tissues, the number of OTUs observed (97% similarity) range from 68 to 152 and from 151 to 220 in the endospheres of plants from AD and PAT, respectively. These values are in accordance with other studies reporting OTUs values from plant endospheres ranging from 100 to 300^2,28,34,35^. However, a significantly greater number of OTUs (from 450 to 3700) have also been reported in plant endospheres by other authors^29,36,37^.

The Shannon index values ranged from 2.28 to 4.41. and from 2.76 to 4.41 in the endospheres of plants from AD and PAT, respectively. The diversity of bacteria in root and leaf tissues of tree peony (*Paeonia* Sect. *Moutan*) had greater Shannon index values (7 to 9) compared to our study^37^. In contrast, a recent study reported Shannon index values that were similar to those we found (3 to 4) in root and leaf endospheres of groundsel (*Senecio vulgaris* L., Asteraceae), also by using the Illumina platform^34^. Similarly, but by using 454‒pyrosequencing, Correa-Galeote *et al*.^35^ reported Shannon index values of 3 to 4 in the root endosphere of maize cultivated at 3,537 meters above sea level in Perú. Interestingly, the Shannon index values found in endosphere tissues from AD plants were generally lower than those reported in other AD habitats, such as soil, lakes and sediments of flat mats, with Shannon index values ranging from 3 to 9^38–41^. This suggests that endospheres of AD plants may harbor less bacterial diversity compared to other niches in the AD, which is considered oldest and driest place on the Earth^42^.

Interestingly, compared with *G*. *mucronata*, *H*. *pilosella* had high bacterial abundances and diversity at the PAT ecosystem, as determined by qPCR and Shannon index, respectively. *H*. *pilosella* is an exotic weed at the Chilean Patagonia, which recognized by its explosive expansion in Patagonian grasslands, often replacing forage plants with the concomitant economic loss for livestock and soil degradation by overgrazing^43^. Therefore, the higher abundance and diversity of endophytic bacteria in *H*. *pilosella* might give this plant species a competitive advantage against other Patagonian plants. Thus, alteration of the composition and activity of endophytic bacteria may be a useful strategy for biological control of invasive plants. However, our study is limited to few sampled plants and major efforts are required to validate this statement and evaluate the potential biocontrol of *H*. *pilosella* expansion in Chilean Patagonia grasslands.

Our Illumina-based analyses revealed the dominance of members of the phyla *Proteobacteria*, *Firmicutes*, *Actinobacteria* and *Bacteroidetes* in the endosphere. It was previously reported that *Proteobacteria* and *Firmicutes* are common inhabitants of plant endospheres with relative abundances ranging from 39% to 97% and 14% to 44%, respectively^4,28,36^. Studies done to analyze root and leave endosphere tissues have also shown a great dominance (over 86%) by the *Proteobacteria*, *Firmicute* and other phyla (such as *Bacteroidetes*, *Acidobacteria* and *Actinobacteria*) in olive trees, peony and groundsel^29,34,37^. Interestingly, a high relative abundance (31%) of *Euryarchaeota* was found in roots of *D*. *spicata*. Member of *Euryarchaeota* have also been found colonizing the endospheres of Mediterranean olive trees^29^ and compartments (rhizosphere, endosphere and phyllosphere) of a halophyte plant (*Salsola stocksii*) from Pakistan^44^.

Relative to what was seen in tissues from the AD and PAT as minor taxa, both ecosystems had a great diversity, represented by members of the *Lentisphaerae*, *Chloroflexi*, *Verrucomicrobia*, *Elusimicrobia*, *Fusobacteria*, *Spirochaetes* and *Acidobacteria*. Low numbers of sequences of these phyla were observed by Hardoim *et al*.^4^, when endophytic data sets from all peer–reviewed publications were revised in the ISI Web of Science and PubMed databases. Similarly, our analyses interestingly revealed the presence of members of the phylum *Cyanobacteria* in roots and leaves of AD plants. High abundances (24% to 47%) of members of this phyla have also been reported in the endospheres of grasses (*Spartina alterniflora*) and mangrove (*Kandelia obovata*)^45^. Lower abundances (1.7%) of *Cyanobacteria* have also been reported in the endosphere and other compartments (rhizosphere and rhizoplane) of the medicinal perennial plant *Stellera chamaejasme* L.^46^. It is noteworthy that *Cyanobacteria* are a diverse group of photosynthetic bacteria (some of them nitrogen‒fixing) that live in a great variety of extreme environments, including soils and rocks from the AD and hot springs and lakes from Argentine Patagonia^42,47,48^. However, and to our knowledge, there have been no previous reports of *Cyanobacteria* associated with the endosphere of plants in AD and PAT.

At the family level, bioinformatic analyses indicated that there was high diversity in the endosphere of plants from both ecosystems, highlighting some dominant groups as the *Halobacteriaceae*, *Bacillaceae*, *Nocardiosaceae* and *Halomonadaceae* in AD plants, and *Pseudomonaceaea* in *G*. *mucronata* in PAT. *Halobacteriaceae* and *Halomonadaceae* are phylogenetically diverse groups of *Archaea* and *Eubacteria* able to survive and proliferate in hypersaline habitats, such as soils from AD^49,50^, and including some endophytes, such as *Euryarchaeota*, *Kushneria endophytica*, *Salinicola tamaricis*^44,51–53^. Similarly, *Bacillaceae* and *Pseudomonaceae* families are commonly found as inhabitant of plant endospheres^34,47,54^. It is noteworthy that the contrast in the family diversity among AD and PAT plants reveals significant differences in the composition of endophytic bacterial communities between locations.

A recent study postulated that plant species are able to recruit specific endophytic bacterial communities^2,55^. Similarly, Gadhave *et al*.^56^ reported that soil inoculation with *Bacillus* spp. modified the diversity, evenness, and community composition of endophytic bacterial in roots of broccoli. However, the composition of endophytic bacterial communities might not only to be ruled by biotic factors (such as plant genotype or competition with other microbes), but by abiotic factors including climate (temperature and drought)^57^ and soil cultivation history^35^. In this context, significant differences between plant species and locations were also observed in rhizosphere bacterial communities in plants from the AD, PAT and Antarctic^14^. In addition, differences in endophytic bacterial community composition may be due to a great portion of unique OTUs (93 to 234 and 195 to 341 in AD and PAT endospheres, respectively) compared with the shared OTUs (53 and 115 in AD and PAT endospheres, respectively) between the plant species as revealed by diagram Venn. Bacteria belonging to the *Firmicutes*, *Proteobacteria*, *Actinobacteria* and *Bacteroidetes*, were mainly observed among the unique OTUs observed in plants. The low number of shared OTUs, compared with unique OTUs, is in contrasts with observations in tree peony grass and mangrove, where Venn analysis showed overlap patterns of OTUs between endophytic bacteria in root and leaves samples^37,45^. Therefore, our results suggest that endosphere of the plant species we studied in each Chilean extreme environment harbored specific bacterial communities unique to their location and plant genotype (species), where *Bacillaceae* and *Enterobacteriacea* could serve as keystone taxa as revealed our LDA analysis.
